# Prehabilitation in a Periprosthetic Fracture of the Femur: A Case Report

**DOI:** 10.7759/cureus.55872

**Published:** 2024-03-09

**Authors:** Shruti S Bhoge, Pratik Phansopkar

**Affiliations:** 1 Musculoskeletal Physiotherapy, Ravi Nair Physiotherapy College, Datta Meghe Institute of Higher Education and Research, Wardha, IND

**Keywords:** periprosthetic fracture, prehabilitation, treatment, pre-operative rehabilitation, pre-operative physiotherapy, rehabilitation, case report, physiotherapy

## Abstract

Periprosthetic fractures (PPF) are related to orthopaedic implants like internal fixators, replacement devices, etc. In this case report, we discussed a 55-year-old male patient who came to our tertiary care hospital with complaints of pain and swelling over the left hip for six months. After radiological investigations, he was diagnosed with a left PPF of the femur with posterior dislocation. He was referred to the musculoskeletal physiotherapy department for in-patient rehabilitation before surgery. He received strengthening exercises for lower limb, back, and abdominal muscles, pain management, gait training, etc. for two weeks before his decided surgery date. The patient showed improved strength and maintained his range. There were an improved Visual Analogue Scale (VAS) score and a Lower Extremity Functional Scale (LEFS) score, which signified a reduction in pain and improved functional independence due to enhanced lower limb function, respectively.

## Introduction

Fractures associated with internal fixators or replacement devices are known as periprosthetic fractures (PPF) [1,2]. According to a study in 2020, the incidence of PPF of the hip after a total hip replacement (THR) is estimated to be 0.1-18% [3]. The incidence rate of PPF of the femur after bipolar hemiarthroplasty was found to be as high as 3.8% in the elderly [4]. Osteopenia, osteoporosis, age above 65, cementless fixation, low-energy trauma, etc. are some of the causes of PPF of the femur [5]. The Vancouver classification is utilised for situating the fracture based on location. According to this classification, type A fractures are associated with fractures of the trochanteric area, type B fractures are related to the stem region, and type C fractures are situated beyond the tip of the stem [6].

This type of fracture is usually surgically treated. For Vancouver type B, the surgical treatment encompasses open reduction and internal fixation (ORIF) or revision arthroplasty. Revision arthroplasty is suggested when there is a stable femoral component, but it is avoided in older patients as it can be an extensive surgery [7]. In those situations and for patients with extreme bone loss, ORIF with allograft strut is considered another way of management [8].

Prehabilitation refers to the rehabilitation of a patient before he undergoes a surgical procedure [9]. Pre-operative physiotherapy is shown to improve recovery periods, pain, functional independence, early discharge, and maintain function [10,11]. In this case report, we will be discussing pre-operative rehabilitation in a case of PPF of the left femur with posterior dislocation.

## Case presentation

Patient information

A 55-year-old male was apparently alright six months ago when he gradually started experiencing pain in the left hip. The pain was gradual in onset, dull aching in nature, aggravated on movement, and relieved on rest. In the last eight days, this pain and swelling were aggravated, and the patient started having difficulty walking. For this complaint, the patient visited our tertiary care hospital where necessary investigation, like an X-ray, was done, which revealed Vancouver type B PPF of the proximal femur with posterior dislocation. The patient gave a previous history of neck of the femur fracture following a two-wheeler collision 15 years back. He was taken to a local hospital, where he underwent uncemented bipolar hemiarthroplasty of the left side. The patient showed no relevant history of diabetes, hypertension, etc. He was referred to musculoskeletal physiotherapy for in-patient pre-operative rehabilitation.

Clinical findings

After taking informed consent, the patient's examination was done in a supine lying position. On inspection, the swelling was visible over the upper and middle region of the left thigh. Over the medial aspect of the left thigh, blackish discolouration was present. On palpation, grade 2 tenderness (patient complains of pain and winces) was present over the left anterior superior iliac spine and greater trochanter of the left femur. In a posteromedial aspect of the mid-thigh, a hard swelling was palpable. The telescopy test was positive, which interpreted dislocation of the head of the femur. The patient's limb girth measurement at thigh level (Table 1) showed increased girth of the left upper thigh.

**Table 1:** Limb girth measurement findings for the upper thigh cm: centimetres

Upper thigh levels	Right (in cm)	Left (in cm)
Proximal	38.8	42.2
Middle	36.3	40.7
Distal	35.2	37.8

Distal pulses like the popliteal artery and dorsalis pedis artery were palpable. There was no alteration in the sensation of the affected lower limb, indicating an intact sensory nervous system. On the Visual Analogue Scale (VAS), the patient rated his pain 6.8 cm on activity and 4.7 cm on rest. The patient had difficulty standing, walking, climbing stairs, donning and doffing lower extremities, and toileting activities. His Lower Extremity Functional Scale (LEFS) score before rehabilitation was found to be 20, which indicated he was having severe limitations. Table 2 and Table 3 show the pre-rehabilitation range of motion (ROM) and manual muscle testing (MMT) findings, respectively.

**Table 2:** Pre-rehabilitation ROM findings (first day) ROM: range of motion

Joint	Movement	Normal	Left	Right
Hip	Flexion	0-120°	0-80°	0-110°
	Extension	0-30°	0-15°	0-26°
	Abduction	0-45°	0-20°	0-40°
	Adduction	0-30°	0-10°	0-26°
	Internal rotation	0-45°	0-25°	0-45°
	External rotation	0-45°	0°	0-45°
Knee	Flexion	0-135°	0-130°	0-128°

**Table 3:** Pre-rehabilitation MMT findings (first day) 2: Full ROM in gravity-eliminated position. 3-: Initiates some but not complete ROM against gravity position. 3: Full ROM against gravity position. 4: Full ROM against minimal resistance. 5: Full ROM against maximal resistance MMT: manual muscle testing; ROM: range of motion

Joint	Muscles	Left	Right
Hip	Flexors	3-/5	4/5
	Extensors	3-/5	4/5
	Abductors	2/5	4/5
	Adductors	2/5	4/5
Knee	Flexors	3/5	4/5
	Extensors	3/5	4/5

Radiological findings

**Figure 1:**
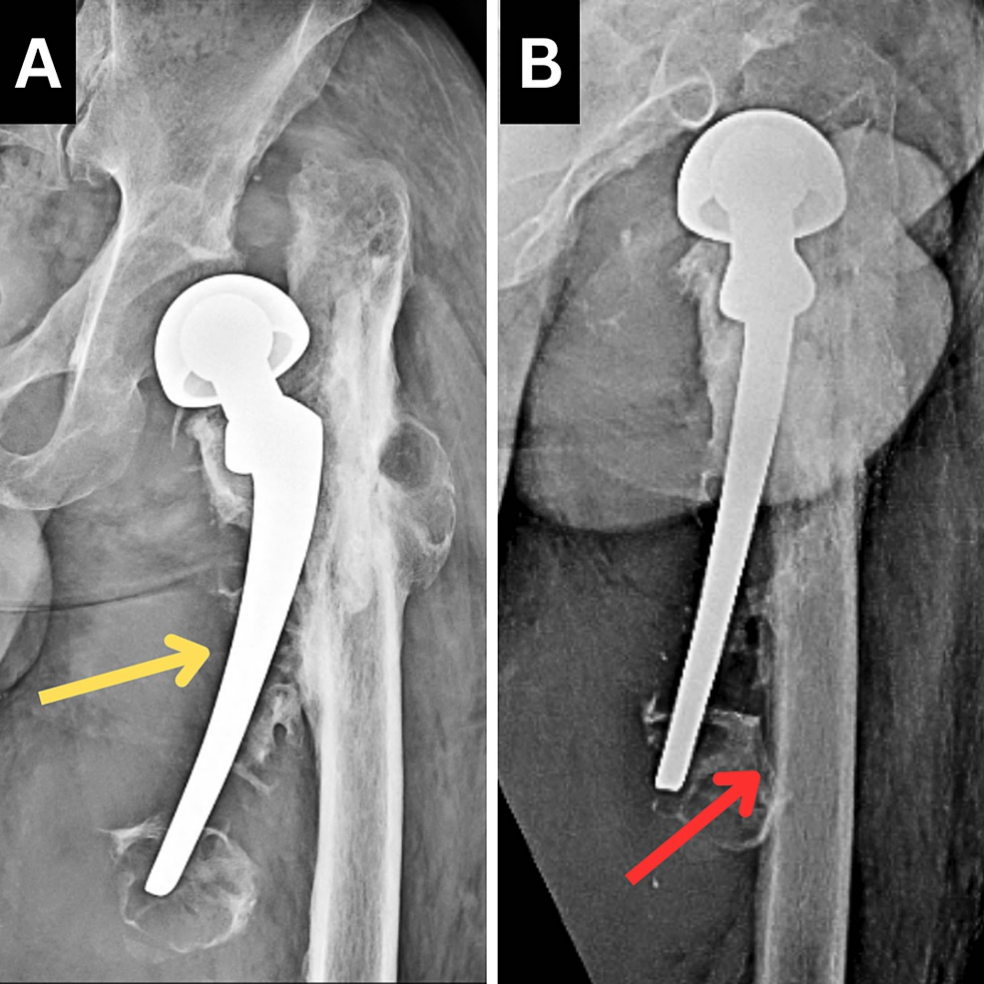
Pre-operative X-ray of the left hip joint A: Anteroposterior view. B: Lauenstein view The yellow arrow indicates PPF of the left femur The red arrow indicates posterior dislocation of the left hip PPF: periprosthetic fracture

The radiological investigation included an X-ray, which was done in an anteroposterior view and Lauenstein view. The X-ray revealed Vancouver type B PPF of the left femur with posterior dislocation. Figure 1 shows the patient's X-ray.

Therapeutic intervention

The patient was referred to the musculoskeletal physiotherapy department for in-patient pre-operative rehabilitation. The surgery was scheduled after 15 days from the day of admission to the hospital. The patient received physiotherapy for 15 days, and every exercise was performed two times a day, i.e., from the day of admission till the day before the scheduled operation. Table 4 summarises the physiotherapeutic intervention received by the patient.

**Table 4:** Pre-operative physiotherapy protocol reps: repetitions; secs: seconds; N/A: not applicable; AROM: active range of motion; ROM: range of motion; DVT: deep vein thrombosis; HAP: hospital-acquired pneumonia

Goals	Intervention	Regimen
Patient education	The patient was informed of his state, the surgery to be performed, and the pertinence of pre- and post-operative physiotherapy treatment. He was also made aware of the advancement of treatment. He was informed about the precautions to be taken	N/A
To reduce pain and swelling	The icing was done on the pain site	For 7-10 minutes
To maintain and enhance strength of lower extremity muscles	Static strengthening exercises for gluteals, quadriceps, hamstrings, and hip abductors and adductors of the left lower extremity	10 reps × 1 set. Each contraction is held for 15 secs
	Dynamic strengthening exercises for right hip musculature as well as bilateral knee muscles	10 reps × 1 set. Each contraction is held for 10 secs
To improve the strength of musculature of the abdomen and back	Static strengthening exercises for back and abdominal muscles	10 reps × 1 set. Each contraction is held for 10 secs, progressing to 15 secs
	Unilateral pelvic bridging	10 reps × 1 set. Each contraction is held for 10 secs
To maintain the ROM of the lower limb	AROM exercises up to the available ROM for the left hip and full ROM exercises for the right hip and bilateral knees	10 reps × 1 set, progressing to 2 sets
To prevent secondary complications like DVT, HAP, etc.	Ankle pumps	10 reps × 1 set
	Breathing exercises	10 reps × 1 set
To improve gait	The patient was having difficulty walking, so the patient was taught non-weight-bearing gait training using a walker	N/A

Follow-up and outcome measures

After 15 days of rehabilitation, ROM and MMT were measured again. Patient outcome measures like VAS and LEFS were measured after 15 days of rehabilitation, which is mentioned in Table 5.

**Table 5:** Findings of outcome measures VAS score is measured by using a 10 cm line on which the patient rates their pain score and then the therapist measures it with a ruler. In VAS, 0 stands for "no pain" and 10 stands for "extremely severe pain" LEFS score is calculated by adding scores of all the components in this scale. Each component is scored using a 5-point Likert scale (0-4) VAS: Visual Analogue Scale; LEFS: Lower Extremity Functional Scale; cm: centimetres

Outcome measures		Day 1	Day 15
VAS	On activity	6.8 cm	5.7 cm
	On rest	4.7 cm	3.3 cm
LEFS		20	38

There was an improvement in the strength of muscles of the right lower extremity as well as the left knee. The strength of the left hip musculature was slightly improved at the end of day 15. The ranges of all joints were sustained. The patient was also able to ambulate with the help of a walker, which helped improve his daily living activity. Table 6 shows day 1 and day 15 findings of left hip ROM and MMT.

**Table 6:** Findings of ROM and MMT (days 1 and 15) 2: Full ROM in gravity-eliminated position. 3-: Initiates some but not complete ROM against gravity position. 3: Full ROM against gravity position. 4: Full ROM against minimal resistance. 5: Full ROM against maximal resistance ROM: range of motion; MMT: manual muscle testing

ROM	Movement	Day 1	Day 15
Hip	Flexion	0-80°	0-83°
	Extension	0-15°	0-18°
	Abduction	0-20°	0-24°
	Adduction	0-10°	0-10°
	Internal rotation	0-25°	0-25°
	External rotation	0°	0°
MMT	Muscles	Day 1	Day 15
Hip	Flexors	3-/5	3/5
	Extensors	3-/5	3/5
	Abductors	2/5	3-/5
	Adductors	2/5	3-/5
Knee	Flexors	3/5	4/5
	Extensors	3/5	4/5

## Discussion

In this case report, we focused on pre-operative rehabilitation in a rare case of PPF of the femur. The number of studies on physiotherapy rehabilitation in these conditions seems to be lacking. Pre-operative rehabilitation has become an integral part of the healthcare system in order to improve postoperative outcomes and prevent any further complications [12]. According to Ackerman and Bennell, the benefits of pre-operative physiotherapy couldn't be confirmed as the sole benefiting factor for the patients improving functional outcomes. They concluded that pre-operative physiotherapy was not successful in improving outcomes in total hip as well as knee replacement [13]. However, a recent study conducted in 2021 by Dangor et al. established that pre-operative rehabilitation led to improved functional independence, reduced the burden on the nursing staff, and led to early discharge [14]. We also saw enhanced functional independence in our patient, as previously, he was even having difficulty walking. A quasi-experimental study by Eldawati and Nurjanah suggested that pre-operative muscle strengthening of a lower extremity with a fracture facilitates early ambulation and recovery post-surgery [15].

In 2020, Vaidya et al. conducted a case study on post-operative rehabilitation of a revised THR following a PPF. During the first week, the rehabilitation focused on pain reduction, maintaining the strength of hip musculature strength, preventing the formation of contractures, and enhancing the hip joint ROM. By the end of the fourth week, the patient was standing by the side of the bed as well as walking around with the support of a walker. He started partial weight-bearing, standing, and walking with a walker in the third month of rehabilitation. This four-month rehabilitation program improved the patient's lower extremity function as well as enhanced his functional independence [16].

## Conclusions

In this case report, we studied the case of a 55-year-old male patient with a PPF of the left femur with posterior dislocation. The area of focus of this case report was pre-operative rehabilitation and its outcome. The patient received a 15-day in-patient rehabilitation before his scheduled date of surgery. His treatment protocol included strengthening exercises, gait training, pain management, etc. The patient showed improved strength in the left lower extremity, and the ROM was also maintained. He was also able to walk with the assistance of a walker.
